# Spatiotemporal immunolocalisation of REST in the brain of healthy ageing and Alzheimer’s disease rats

**DOI:** 10.1002/2211-5463.13036

**Published:** 2020-12-01

**Authors:** Myrthe Mampay, María Velasco‐Estevez, Sara O. Rolle, Aisling M. Chaney, Hervé Boutin, Kumlesh K. Dev, Emad Moeendarbary, Graham K. Sheridan

**Affiliations:** ^1^ School of Pharmacy and Biomolecular Sciences University of Brighton UK; ^2^ Drug Development Department of Physiology School of Medicine Trinity College Dublin Ireland; ^3^ The Sainsbury Welcome Centre for Neural Circuits and Behaviour University College London UK; ^4^ Faculty of Biology, Medicine and Health School of Health Sciences Division of Informatics, Imaging and Data Sciences University of Manchester UK; ^5^ Wolfson Molecular Imaging Centre University of Manchester UK; ^6^ Division of Neuroscience and Experimental Psychology Faculty of Biology, Medicine and Health School of Biological Sciences University of Manchester UK; ^7^ Department of Mechanical Engineering University College London UK; ^8^ School of Life Sciences Queens Medical Centre University of Nottingham UK; ^9^ Present address: Spanish National Cancer Research Centre (CNIO) Madrid Spain; ^10^ Present address: Department of Radiology Stanford University Palo Alto CA 94304 USA

**Keywords:** ageing, Alzheimer’s disease, hippocampus, REST, synapse, TgF344‐AD rats

## Abstract

In the brain, REST (Repressor Element‐1 Silencing Transcription factor) is a key regulator of neuron cell‐specific gene expression. Nuclear translocation of neuronal REST has been shown to be neuroprotective in a healthy ageing context. In contrast, inability to upregulate nuclear REST is thought to leave ageing neurons vulnerable to neurodegenerative stimuli, such as Alzheimer’s disease (AD) pathology. Hippocampal and cortical neurons are known to be particularly susceptible to AD‐associated neurodegeneration. However, REST expression has not been extensively characterised in the healthy ageing brain. Here, we examined the spatiotemporal immunolocalisation of REST in the brains of healthy ageing wild‐type Fischer‐344 and transgenic Alzheimer’s disease rats (TgF344‐AD). Nuclear expression of REST increased from 6 months to 18 months of age in the hippocampus, frontal cortex and subiculum of wild‐type rats, but not in TgF344‐AD rats. No changes in REST were measured in more posterior cortical regions or in the thalamus. Interestingly, levels of the presynaptic marker synaptophysin, a known gene target of REST, were lower in CA1 hippocampal neurons of 18‐month TgF344‐AD rats compared to 18‐month wild‐types, suggesting that elevated nuclear REST may protect against synapse loss in the CA1 of 18‐month wild‐type rats. High REST expression in ageing wild‐type rats did not, however, protect against axonal loss nor against astroglial reactivity in the hippocampus. Taken together, our data confirm that changes in nuclear REST expression are context‐, age‐ and brain region‐specific. Moreover, key brain structures involved in learning and memory display elevated REST expression in healthy ageing wild‐type rats but not TgF344‐AD rats.

## Graphical abstract

Upregulation of the transcription factor, REST, is neuroprotective in the ageing brain. We quantified changes in nuclear REST expression in the brains of ageing wild‐type (WT) and Alzheimer’s disease (AD) rats. REST increased in structures involved in learning and memory in WT but not AD rats, potentially explaining why these regions are vulnerable to synapse loss, neuroinflammation and astrogliosis.

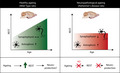

**Abbreviations**
AD: Alzheimer’s Disease
a.f.u: Arbitrary fluorescence units
APP: Amyloid precursor protein
Aβ_1–42_: Amyloid beta‐peptide fragment 1–42
CDK5R1: Cyclin‐dependent kinase 5 Regulatory Subunit 1
DAPI: 4′,6‐diamidino‐2‐phenylindole
DG: dentate gyrus
FADD: FAS‐associated death domain protein
fMRI: functional Magnetic Resonance Imaging
GFAP: glial fibrillary acidic protein
Grin2A: Glutamate Ionotropic Receptor NMDA Type Subunit 2A
HCN1: Hyperpolarisation activated Cyclic Nucleotide gated potassium channel
HPA: Hypothalamic‐pituitary‐adrenal axis
L1CAM: Neural cell adhesion molecule L1 precursor
LPS: lipopolysaccharide
MAPKII: MAP kinase‐activated protein kinase 2
MCI: mild cognitive impairment
MPP+: 1‐methyl‐4‐phenylpyridinium
MPTP: 1‐methyl‐4‐phenyl‐1,2,3,6‐tetrahydropyridine
NFH: neurofilament heavy chain
NRSF: neuron‐restrictive silencer factor
PBS: phosphate‐buffered saline
PSEN1: presenilin‐1
PUMA: P53 Upregulated Modulator of Apoptosis
REST: RE1‐Silencing Transcription factor
ROI: Region of Interest
ROS: Reactive Oxygen Species
SN: Substantia Nigra
Syp: Synaptophysin
TG: Transgenic
WT: wild‐type

Alzheimer’s disease (AD) is a progressive neurodegenerative disorder characterised by profound memory loss, personality changes, apathy and language impairments [1]. Many genetic risk factors for AD, including the ε4 isoform of apolipoprotein E (ApoE4), have been identified [2]. However, the most profound nongenetic risk factor for AD is ageing and the deterioration of homeostatic physiological systems [3]. In approximately 90% of AD cases, patients are older than 65 years and the incidence of AD doubles with every 5 years over the age of 65 [4]. At the cellular level, classic hallmarks of AD include extracellular amyloid‐β_1–42_ (Aβ_1–42_) plaques, intracellular neurofibrillary tangles of hyperphosphorylated tau, neurodegeneration, loss of synapses and extensive neuroinflammation [4, 5, 6].

The cognitive decline observed in AD patients correlates closely with synaptic dysfunction [7]. Disruption of synaptic plasticity, ultimately resulting in the loss of synapses and neurodegeneration, is regarded as a common pathological event in AD [8, 9, 10], whereas neuronal plasticity is largely preserved in the hippocampal and cortical regions of the healthy ageing brain [10]. As well as synaptic dysfunction, AD is associated with high levels of chronic neuroinflammation [4]. Glial cells can sense and respond to Aβ_1–42_ deposition in hippocampal and cortical brain regions in an attempt to engulf and degrade the toxic peptide aggregates. However, activated glial cells release a cascade of pro‐inflammatory cytokines and chemokines, which exacerbate the pathogenic process [11]. Moreover, it has been suggested that AD‐associated neuroinflammation may contribute more to the pathogenesis of the disease, compared to the impact of amyloid‐β plaques and tau tangles themselves [12].

Recently, the transcriptional repressor REST (RE1‐silencing transcription factor), also known as NRSF (Neuron‐Restrictive Silencer Factor), was shown to play a crucial role in neuroprotection in the ageing brain [13]. REST is a known transcriptional regulator of more than 2000 neuron‐specific target genes that encode for a plethora of proteins involved in synaptic plasticity (e.g., Syp), neurotransmitter receptors (e.g., Grin2A), ion channel formation (e.g., HCN1) and axonal guidance (e.g., L1CAM) [14]. Whilst neuronal REST normally resides inactive in the cytoplasm of differentiated neurons, ‘healthy ageing’ is associated with a significant induction of REST in neuronal nuclei in the prefrontal cortex. In contrast, this age‐related induction of nuclear REST was less apparent in patients with mild cognitive impairment (MCI) and almost completely absent in AD patients [13]. Upregulation of active REST was observed in the nucleus of human prefrontal cortical neurons during healthy ageing and was found to transcriptionally repress genes involved in neuronal cell death (e.g., MAPKII, FADD, PUMA) and AD‐related genes (e.g., presenilin 2, γ‐secretase and CDK5R1), thereby providing age‐related neuroprotection [13]. Furthermore, the ageing‐induced increase in nuclear REST was shown to protect neurons from Aβ_1–42_ pathology and oxidative stress [13, 15, 16]. In contrast, in human neuropathological diseases, including AD and Parkinson’s disease which are both hallmarked by protein misfolding and aggregation, REST does not translocate to neuronal nuclei to exert its protective functions [13, 16].

These findings suggest that nuclear REST expression confers neuroprotection in the ageing brain. However, REST‐dependent gene regulation is highly cell‐type specific, as well as brain region and context‐dependent [17]. On one hand, nuclear translocation of REST in the hippocampus of healthy ageing humans was shown to be neuroprotective [13]. On the other hand, REST nuclear translocation in response to ischaemia and seizures has been linked to the death of hippocampal neurons [14]. In Huntington’s disease, aberrant accumulation of REST occurs in the nuclei of vulnerable striatal neurons [18]. Whilst in Parkinson’s disease, nuclear translocation of REST does not occur in dopaminergic neurons of the substantia nigra (SN) or neocortex [16]. These apparently opposing actions of REST are analogous to the complex role of various cytokines in the brain. For example, relatively high concentrations of cytokines released for chronic time‐periods can be detrimental, but lower concentrations present for acute periods can potentiate neuronal function [19]. Overall, the transcription factor REST is a promising therapeutic target in AD and other neurodegenerative diseases. However, very little is known about ageing‐associated changes in REST expression and distribution patterns throughout the healthy ageing brain and the Alzheimer’s diseased brain.

Therefore, we investigated the spatiotemporal localisation of REST in healthy ageing and Alzheimer’s disease rat brains. The aim was to visualise changes in nuclear REST expression throughout the brain of healthy ageing rats and to contrast this with a transgenic model of AD. The TgF344‐AD rat model [20] overexpresses human amyloid precursor protein (APP) with the Swedish mutation (APP_SWE_) and mutated human presenilin‐1 (PSEN1ΔE9). REST expression was quantified by immunofluorescence in brain sections from wild‐type (WT) Fischer‐344 and transgenic (TG) rats. We focused on brain regions involved in learning and memory which are affected by AD pathology, including the hippocampus and cortical regions. To investigate the potential relationship between REST levels and pathological hallmarks of AD, we also quantified changes in the abundance of protein and peptide markers associated with amyloid plaques (Aβ_1–42_), synaptic dysfunction (synaptophysin), age‐related neuronal atrophy (neurofilament‐H) and neuroinflammation‐induced astrogliosis, that is, glial fibrillary acidic protein (GFAP).

## Materials and methods

### Ethics statement

All experiments involving animals and Schedule 1 protocols used to obtain brain tissue were approved by the Animal Welfare and Ethical Review Body (AWERB committee) of the University of Manchester. This study was conducted in accordance with the principles of the Basel Declaration and adhered to the legislation detailed in the UK Animals (Scientific Procedures) Act 1986 Amendment Regulations (SI 2012/3039). All efforts were taken to maximise animal welfare conditions and to reduce the number of animals used in accordance with the European Communities Council Directive of 20 September 2010 (2010/63/EU).

### Fischer‐344 and TgF344‐AD rats

Two male and two female wild‐type (WT) Fischer‐344 and TgF344‐AD (TG) rats with the APP_SWE_ and PSEN1ΔE9 mutations were purchased from Prof T. Town laboratory (University of Southern California, USA) and were set up as breeding pairs in‐house at the Biological Services Unit (BSU) in the University of Manchester, UK. TG rats and WT littermates were separated into different cages for the ageing studies. For immunohistological assessment, the number (*n*) of rats per group is detailed in the corresponding figure legend. All animals used were male and were housed in groups of two to four per cage with individual ventilation, environmental enrichment and access to food and water *ad libitum*. A 12‐h light/dark cycle was used, with light from 7 am until 7 pm, in a holding room maintained at 21 ± 1 °C and ~ 55% relative humidity.

### Tissue sectioning

TgF344‐AD and WT rats were euthanised using an isoflurane overdose, confirmed by cervical dislocation. The brains were collected, snap‐frozen using isopentane on dry ice and stored at −80 °C. Sagittal brain sections (20 µm thick) between 1 mm and 3.36 mm lateral to Bregma were cut using a cryostat (Leica CM3050s, Leica Biosystems, Germany) and stored at −80 °C. Prior to immunofluorescence, frozen sections were removed from the −80 °C freezer and allowed to air dry at 22 °C for 20 min before fixation in 70% ethanol for 30 min. They were then permeabilised in 0.2% Triton‐X/PBS for 30 min, followed by several PBS washes, and then blocked with 5% BSA in PBS for 1.5 h at 22 °C.

### Immunohistochemistry

Sections from WT Fischer‐344 and TgF344‐AD rats were incubated overnight at 22 ± 2 °C with the following primary antibodies, (1) 1 : 1000 dilution of rabbit anti‐amyloid β_1–42_ (mOC98) (ab201061, RRID: AB_2722492, Abcam, Cambridge, UK), a conformation‐specific antibody that recognises a discontinuous epitope of Aβ that maps to segments AEFRHD and EDVGSNK; (2) 1 : 1000 dilution of chicken antiglial fibrillary acidic protein (GFAP) (ab4674, RRID: AB_304558, Abcam); (3) 1 : 500 dilution of rabbit anti‐REST (ab202962, Abcam); (4) 1 : 5000 dilution of chicken antineurofilament heavy chain (NFH) (ab5539, RRID: AB_11212161, Merck Millipore, Watford, UK); (5) 1 : 2000 dilution of mouse anti‐synaptophysin (7H12) (9020S, RRID: AB_2631095, Cell Signalling, Leiden, The Netherlands). Slides were then washed in PBS and incubated for 4 h at 22 °C with the following secondary antibodies, (1) donkey anti‐rabbit 555 (SAB4600061, Merck, Darmstadt, Germany), (2) donkey anti‐chicken IgY (H + L) CF™ 633 (SAB4600127, Merck) and (3) donkey anti‐mouse Alexa 555 (ab150110, Abcam). Finally, slides were washed, and cover slipped using ProLong^®^ Gold antifade mounting medium with DAPI (P36935, Thermofisher Scientific, Cambridge, UK).

### Microscopy

All images of REST, Aβ_1–42_ peptide, GFAP, NFH and synaptophysin (Syp) expression in sagittal cryosections were captured using an Axio Scan.Z1 slide scanner (Zeiss, Munich, Germany) with a 20× magnification objective (Plan‐Apochromat). Individual images were montaged together automatically in ZEN software (Zeiss) to reconstruct a single image of the whole brain section. For quantitative fluorescence intensity analysis, all images were captured in a single uninterrupted run (~50 h imaging time) and uniform microscope settings were maintained throughout the session. The images were exported as 8‐bit.tif files for fluorescence intensity quantification.

### Image analysis of amyloid plaque expression

Image analysis for Aβ_1–42_ was conducted using the software package ImageJ [21]. Briefly, Aβ_1–42_ fluorescence intensity was quantified by manually selecting 10 regions of interest (ROI) for each brain structure and calculating the average fluorescence intensity within each ROI. Aβ_1–42_ morphological analysis was performed using the particle analyser command in ImageJ software [22]. Images were thresholded and made binary, holes were filled, and particles were analysed showing the number of particles per area and the perimeter. The following sequence of commands was used: *Open image > Threshold > Make binary > Fill holes > Outline > Analyse particles*. Data are presented as the average number of plaques per mm^2^, the average perimeter of each plaque (µm) and the mean Aβ_1–42_ fluorescence intensity.

### Image analysis of REST expression

Analysis of REST expression was conducted using images captured from two distinct immunolabelled brain sections, with 4–8 animals per age‐group and genotype, as described in the figure legends. The occipital, parietal, cingulate and frontal cortices as well as the thalamus, subiculum and hippocampal CA and dentate gyrus regions were chosen for analysis due to previous reports of significant AD‐like pathology in these brain areas of the TgF344‐AD rat model [20, 23]. The aim was thus to measure changes in nuclear REST expression with age and AD pathogenesis in these regions. In the hippocampus, only regions containing principal neuronal cell bodies (i.e., stratum pyramidale of the CA1 and CA3, and stratum granulosum of the DG) were analysed. Fluorescence channels were split and converted to greyscale. Changes in nuclear REST expression were measured using the open‐source image analysis software, CellProfiler [24]. Briefly, nuclei were automatically detected using DAPI staining as a nuclear marker and the co‐localised intensity of REST fluorescence was measured. Specifically, the following sequence of commands was used: *Import images > Convert Color to Gray > Identify Primary Objects > Identify Secondary Objects > Measure Object Intensity*. Data are presented as mean nuclear REST fluorescence intensity (see Fig. S1 for further details).

### Image analysis of Synaptophysin expression

Analysis of synaptophysin expression in the hippocampus was also performed using two immunolabelled brain sections from 4–8 animals per age‐group and genotype. Mean fluorescence intensities in the CA1, CA3 and dentate gyrus regions were measured using ImageJ software [21, 25]. Briefly, five same‐sized ROIs were drawn within each hippocampal region and the mean fluorescence intensity within each ROI was measured. Data are presented as the average synaptophysin fluorescence intensity per animal for each hippocampal region analysed.

### Image analysis of axonal density and diameter

Quantification of axonal density and diameter was performed using a Hessian‐based feature in ImageJ. NFH‐labelled images from two different brain sections from 4–8 animals per age‐group and genotype were used. Only neurons within the CA1, CA3 and DG regions of the hippocampus were measured for this analysis. Here, the ImageJ software plug‐in, FeatureJ, was used to extract line‐like information through a Hessian‐based filter [26, 27]. The following parameters in the plug‐in for Hessian analysis were selected: (1) Largest eigenvalue of Hessian tensor; (2) Absolute eigenvalue comparison; (3) Smoothing scale factor = 0.5. These settings create an image of the largest eigenvalues after absolute values of Hessian matrices comparison. Next, a line scan profile analysis was performed using the ImageJ ‘Line’ tool by drawing a straight line through each region of interest. Pixel intensity across this line was plotted using the ‘Plot Profile’ tool in ImageJ. All the line scan data were imported to R for further analysis. Here, the mean number of peaks per unit area (mm) was calculated to measure axonal density. To measure changes in axonal diameter, the width of each peak at half‐height were calculated.

### Image analysis of hippocampal astrogliosis

GFAP‐labelled images from two distinct brain sections from 4–9 animals per age‐group and genotype were used. Astrocytic morphology was analysed using the imagej software plug‐in, bonej [28]. Briefly, after thresholding and selecting the hippocampal region of interest (CA1, CA3 or DG), the image was made binary and skeletonised. Data are presented as the number of GFAP‐positive astrocyte branches per area (mm^2^) and the average branch length (µm).

### Statistical analysis

All statistical analysis was performed using graphpad ^®^ prism 7 (RRID: SCR_015807). Assessment of normality was carried out using D’Agostino analysis. To analyse changes in fluorescence intensities, one‐way or two‐way ANOVAs were performed, as appropriate, on the raw fluorescence values, followed by Holm‐Sidak multiple comparisons post hoc tests. Changes in expression were considered significant when the *P* value < 0.05.

## Results

### Amyloid plaque pathology increases with age in the hippocampus of TgF344‐AD rats

**Fig. 1:**
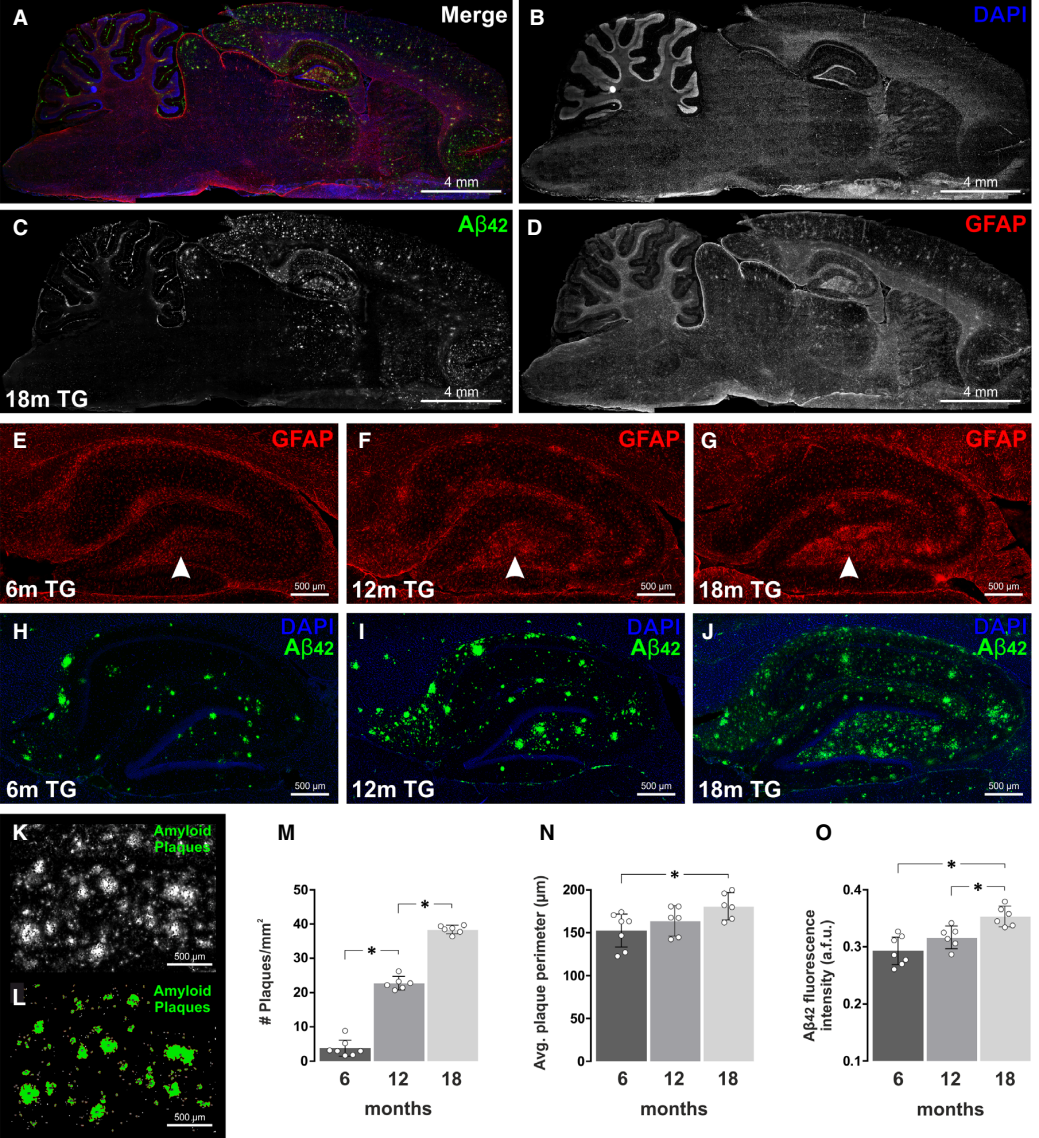
Amyloid plaque pathology increases with age in the TgF344‐AD rat hippocampus. (A) Sagittal section of an 18‐month TgF344‐AD (18m TG) rat brain immunofluorescently labelled using (B) the cell nucleus marker DAPI (blue), (C) a conformation‐specific Aβ_1‐42_ antibody (green), and (D) the astrocyte marker GFAP (red). Scale bar = 4 mm. Extensive amyloid plaque pathology was observed in the hippocampus and neocortex and, to a lesser extent, in the thalamus, striatum and cerebellum. Very few amyloid plaques were visualised in the medulla, pons or ventral midbrain structures, although the inferior colliculus (auditory integration centre) in the dorsal midbrain did show signs of amyloid plaque pathology. Aged TgF344‐AD rats also displayed an increase in astrocyte reactivity. This was particularly evident in the hilus region of the hippocampal dentate gyrus (white arrowheads), which expressed lower levels of GFAP staining in (E) 6m TG rats, compared to (F) 12m TG, and (G) 18m TG. Scale bar = 500 µm. Coinciding with this increase in astrocyte reactivity was an enhanced deposition of Aβ_1‐42_ and accumulation of amyloid plaques in all areas of the hippocampus from 6 to 18 months of age. This is clearly seen in representative images of (H) 6m TG rats, (I) 12m TG and (J) 18m TG. Scale bar = 500 µm. Amyloid plaque pathology was quantified using the particle analysis tool in ImageJ software. (K) Areas of Aβ_1‐42_ staining were thresholded as seen in (L) and the density (M), perimeter (N) and fluorescence intensity (O) of plaques were quantified in 6‐, 12‐ and 18‐month TgF344‐AD rats. Data are presented as the mean ± 95% confidence intervals and analysed using a one‐way ANOVA with Holm‐Sidak post hoc test. * represents a p value < 0.05. The number (*n*) of rats per group were as follows; 6m TG (*n* = 7); 12m TG (*n* = 6); 18m TG (*n* = 6).

We first investigated the gradual increase in amyloid plaque burden in the hippocampus of adult transgenic rats. Sagittal sections of the TgF344‐AD rat brain were cryosectioned and immunolabelled for Aβ_1–42_ (amyloid plaques), as well as GFAP (astrocytes) and the cell nucleus counterstain DAPI (Fig. 1A–D). Extensive plaque pathology was apparent in the hippocampus and neocortex of 18‐month (18m) TgF344‐AD rat brains (Fig. 1C) and to a lesser extent in the thalamus, cerebellum, and striatum. In comparison, very few amyloid plaques were visualised in the medulla, pons or ventral midbrain structures, although the inferior colliculus (auditory integration centre) in the dorsal midbrain did show signs of amyloid plaque pathology. An increase in astrocyte reactivity/hypertrophy was evident from 6 to 18 months of age (Fig. 1E–G), particularly in the hilus region of the dentate gyrus and around amyloid plaques, as described previously by others [29, 30, 31]. Coinciding with this increase in astrocyte reactivity was an enhanced deposition of Aβ1–42 and accumulation of amyloid plaques in all areas of the hippocampus from 6 to 18 months of age. (Fig. 1H–J). Hippocampal amyloid plaque characteristics were quantified using imagej software (Fig. 1K, L). The density (number of plaques per mm^2^), size (average perimeter) and fluorescence intensity of amyloid plaques increased in TgF344‐AD rats from 6 to 18 months of age (Fig. 1M–O).

### REST expression increases with age in the subiculum and frontal cortical regions of wild‐type rats

**Fig. 2:**
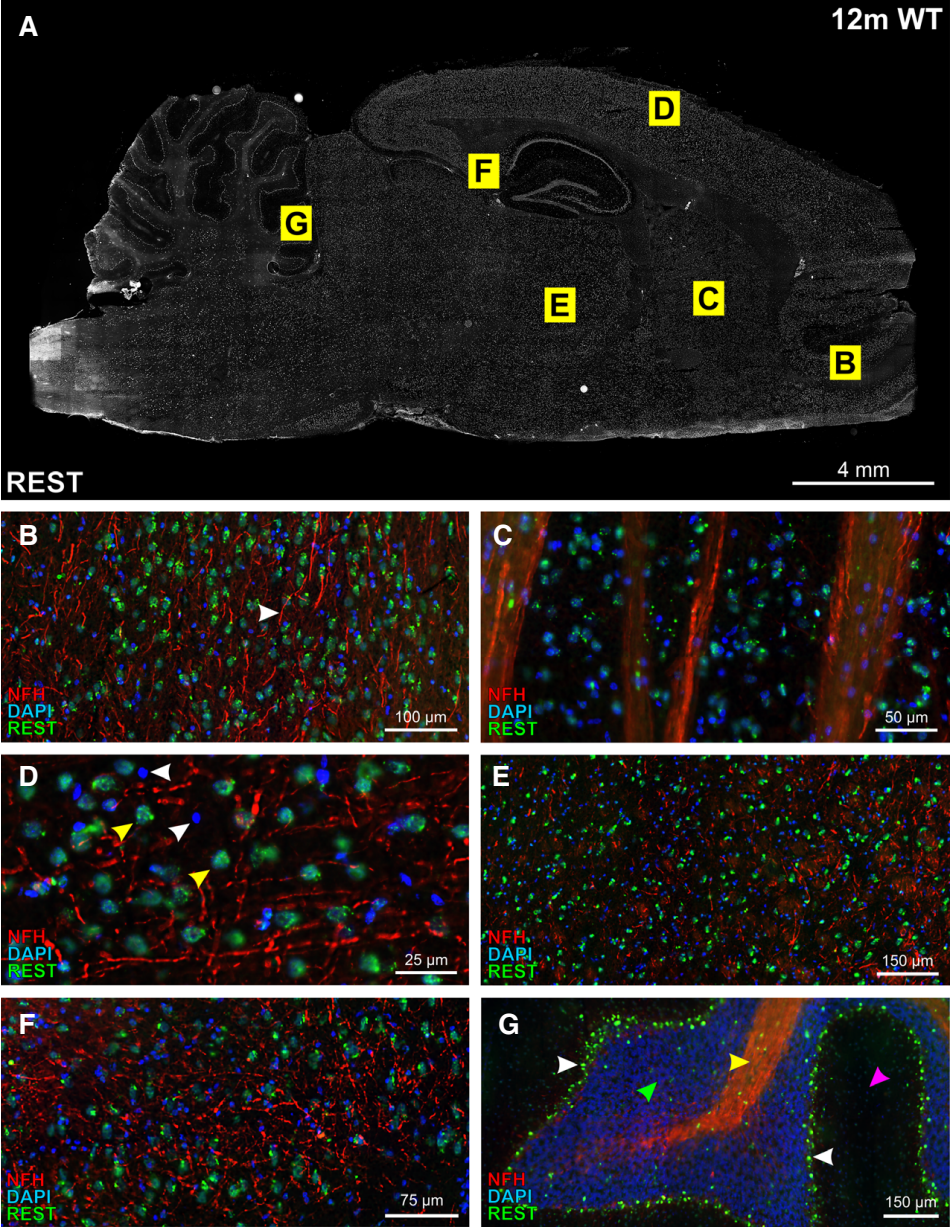
REST localises to the nuclear compartment of cells and is absent from axons. (A) Representative image of REST immunofluorescence (white) in the brain of a 12‐month wild‐type (12m WT) Fischer‐344 rat. Scale bar = 4 mm. (B–G) Zoomed‐in images from the 12‐month WT rat brain depicted in (A). (B) REST expression (green) in the anterior olfactory nucleus. The white arrow points to a neurofilament‐H‐labelled axon devoid of REST staining. Scale bar = 100 µm. (C) REST expression in the striatum. Scale bar = 50 µm. (D) REST expression in the neocortex. Scale bar = 25 µm. REST predominantly localises to the nuclear compartment of cells with large nuclei (yellow arrowhead) and decondensed chromatin (i.e., faint DAPI staining). These cells are more likely to be NeuN‐positive neurons than the small nuclei with condensed chromatin (white arrows), as described in [63]. (E) REST expression in the thalamus. Scale bar = 150 µm. (F) REST expression in the subiculum. Scale bar = 75 µm. Like most areas in the brain, REST is localised to larger nuclei with decondensed chromatin and absent from neuronal axons. (G) REST expression in the cerebellar lobule. Scale bar = 150 µm. There is relatively low expression of REST in the granule cell layer (green arrowhead) and the molecular layer (magenta arrowhead) compared to the Purkinje neuronal nuclei (white arrowheads). The Purkinje axons (arbor vitae; yellow arrowhead) also have low levels of REST.

**Fig. 3:**
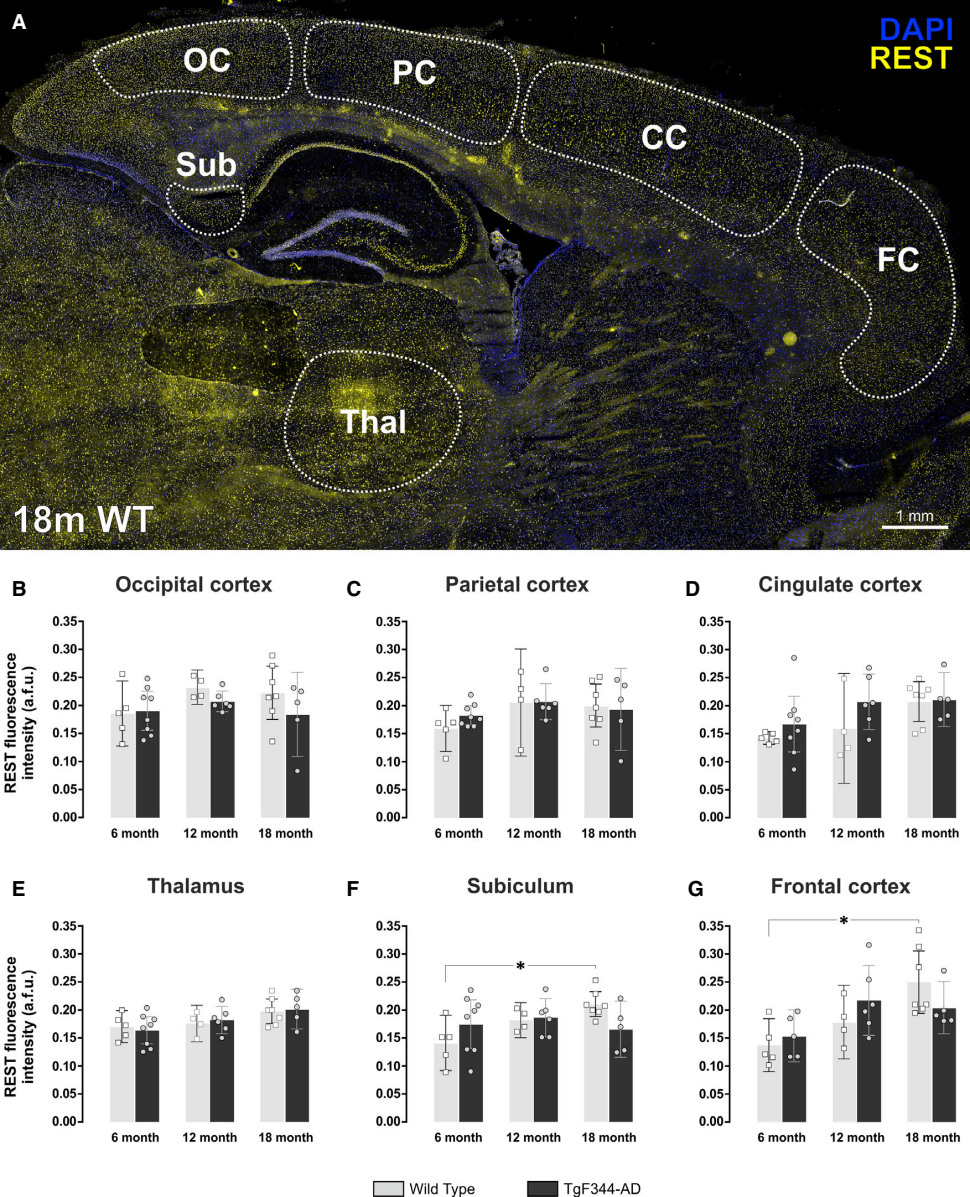
REST increases with age in the subiculum and frontal cortex of wild‐type Fischer‐344 rats. (A) Sagittal section of an 18‐month wild‐type (18m WT) rat brain immunofluorescently‐labelled using the cell nucleus marker DAPI (blue), the axonal marker neurofilament‐H (red), and the transcriptional repressor, REST (green). Scale bar = 1 mm. In the more posterior regions of the neocortex, known as the (B) occipital (OC), (C) parietal (PC), and (D) cingulate cortices (CC), there were no changes in REST expression with age in either WT or TgF344‐AD rats. (E) There were also no changes in REST expression in the thalamus (Thal) in either WT or TgF344‐AD rats from 6 to 18 months. (F) The subiculum (Sub), however, did show elevated REST levels from 6 to 18 months of age, but only in WT rats. (G) Similarly, the frontal cortex (FC) displayed the largest increases in REST expression from 6 to 18 months, but again only in WT rats. Data are presented as the mean ± 95% confidence intervals and analysed using a two‐way ANOVA with Holm‐Sidak post hoc test. *Represents a *P* value < 0.05. The number (*n*) of rats per group was as follows: 6m WT (*n* = 5); 12m WT (*n* = 4); 18m WT (*n* = 7); 6m TG (*n* = 8); 12m TG (*n* = 6); 18m TG (*n* = 5).

Our next aim was to assess the relative expression of REST throughout different regions of the adult rat brain. Wild‐type and TgF344‐AD rat brains (6, 12 and 18 months) were sectioned and immunolabelled for REST (Fig. 2A–G). A representative image from a 12‐month wild‐type rat is shown in Fig. 2A. REST expression was notably higher in grey matter versus white matter regions of the brain. The corpus callosum, for example, displayed low levels of REST expression. Areas of the brain that contained higher levels included the anterior olfactory nucleus (Fig. 2B), striatum (Fig. 2C), neocortex (Fig. 2D), thalamus (Fig. 2E), subiculum (Fig. 2F), Purkinje cells of the cerebellum (Fig. 2G) and principal neurons of the hippocampal cell layers. The pons, medulla and midbrain structures also contained relatively high nuclear REST expression. We first quantified REST fluorescence intensity in DAPI‐labelled nuclei of the neocortex, subiculum and thalamus, areas that showed relatively high expression of amyloid plaque pathology in TgF344‐AD rats. The neocortex was divided into four distinct subregions, that is, the occipital, parietal, cingulate and frontal cortices (Fig. 3A). There were no differences in mean nuclear REST intensities between wild‐type and TgF344‐AD rats. Moreover, there were no changes in REST expression in the thalamus, nor in the occipital, parietal and cingulate cortices with advancing age (Fig. 3B–E). However, REST expression increased from 6 to 18 months in the subiculum (mean = 0.14, 95% CI = [0.09, 0.19] vs 0.21, 95% CI = [0.19, 0.23] arbitrary fluorescence units (a.f.u.), *P* < 0.05) and frontal cortex (mean = 0.14, 95% CI = [0.09, 0.18] vs 0.25, 95% CI = [0.19, 0.31] a.f.u., *P* < 0.05) of wild‐type rats (Fig. 3F, G), but this effect was not observed in TgF344‐AD rats.

### Ageing increases REST expression in the hippocampus of wild‐type rats

**Fig. 4:**
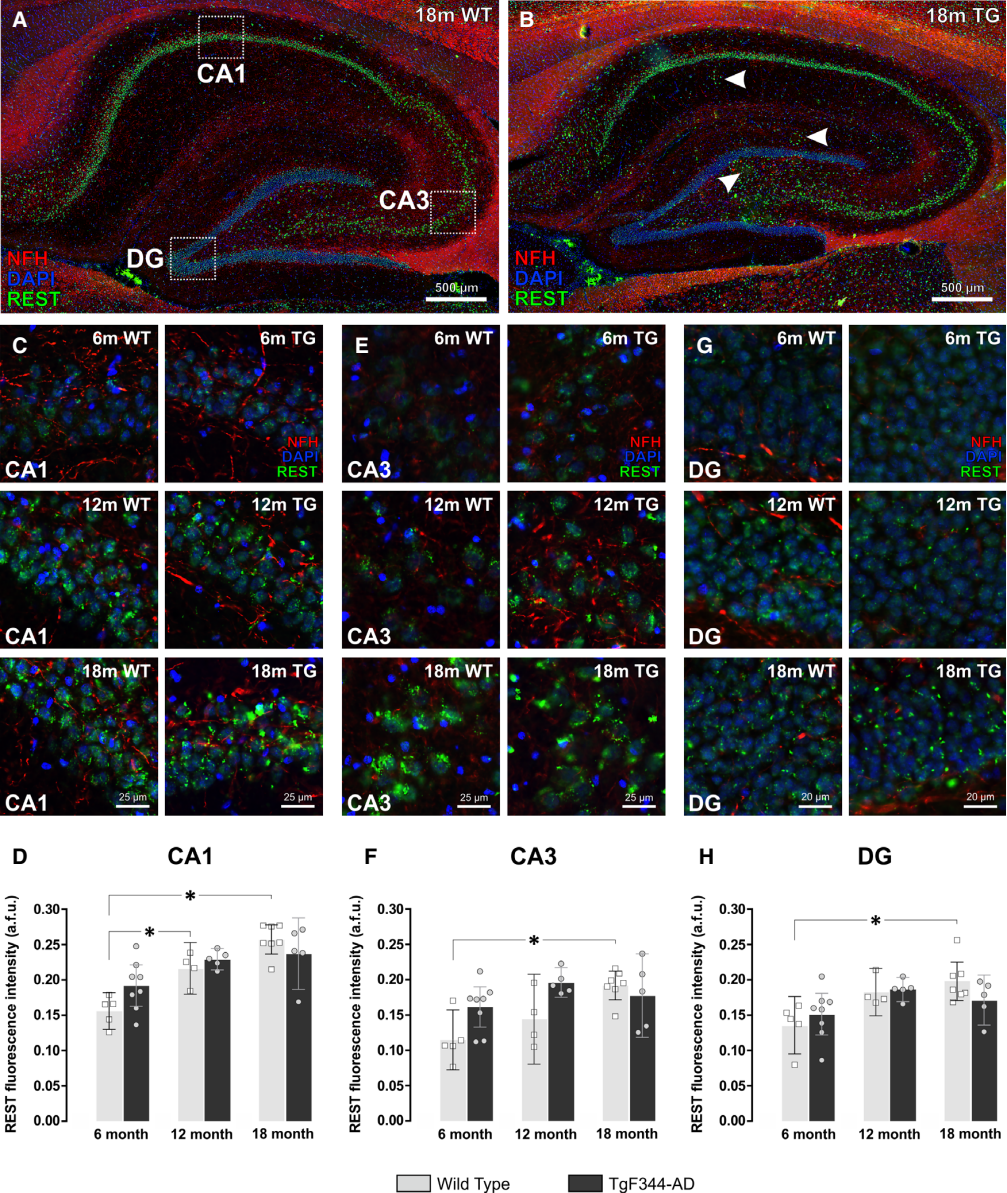
REST increases with age in hippocampal CA1, CA3 and dentate gyrus of wild‐type Fischer‐344 rats. (A) Sagittal section of the dorsal hippocampus of an 18‐month wild‐type (WT) rat and (B) 18‐month TgF344‐AD (TG) rat immunofluorescently labelled using the cell nucleus marker DAPI (blue), the axonal marker neurofilament‐H (red) and the transcription factor, REST (green). Scale bar = 500 µm. Increased REST expression was noted in areas corresponding to amyloid plaque deposition (white arrows). (C) Cropped images showing REST expression in the pyramidal cell layer of the CA1 region in 6‐, 12‐ and 18‐month WT and TG rats. Scale bar = 25 µm. (D) REST expression increased in the CA1 region of WT rats from 6 to 18 months of age, but not in TG rats. (E) Cropped images showing REST expression in the pyramidal cell layer of the CA3 region in 6‐, 12‐ and 18‐month WT and TG rats. Scale bar = 25 µm. (F) REST expression increased in the CA3 region of WT rats from 6 to 18 months of age, but not in TG rats. (G) Cropped images showing REST expression in the granule cell layer of the dentate gyrus (DG) in 6, 12 and 18m WT and TG rats. Scale bar = 20 µm. (H) REST expression increased in the DG region of WT rats from 6 to 18 months of age, but not in TG rats. Data are presented as the mean ± 95% confidence intervals and analysed using a two‐way ANOVA with Holm‐Sidak post hoc test. *Represents a *P* value < 0.05. The number (*n*) of rats per group were as follows; 6m WT (*n* = 5); 12m WT (*n* = 4); 18m WT (*n* = 7); 6m TG (*n* = 8); 12m TG (*n* = 5); 18m TG (*n* = 5).

Nuclear REST expression was also measured in the hippocampal CA1, CA3 and dentate gyrus of wild‐type and TgF344‐AD rats (Fig. 4A, B). Mean REST intensity increased in the CA1 region of wild‐type rats from 6 to 12 months of age (mean = 0.16, 95% CI = [0.13, 0.18] vs 0.22, 95% CI = [0.18, 0.25] a.f.u., *P* < 0.05). The levels of REST remained elevated in 18‐month‐old WT rats (mean = 0.26, 95% CI = [0.24, 0.28] a.f.u.) (Fig. 4C, D). Similarly, although slightly delayed, REST expression increased from 6 to 18 months in the CA3 (Fig. 4E, F) and DG (Fig. 4G, H) regions of wild‐type rats. There were no significant changes in REST expression over time in the hippocampus of TgF344‐AD rats. This appeared to be due to the slightly higher basal levels of nuclear REST in the hippocampus of 6‐month‐old TgF344‐AD rats versus 6‐month wild‐types, coupled with slightly lower REST expression in 18‐month transgenics versus 18‐month wild‐types, although these differences were not statistically significant.

### Late synapse loss in hippocampal CA1 region of 18‐month TgF344‐AD rats

**Fig. 5:**
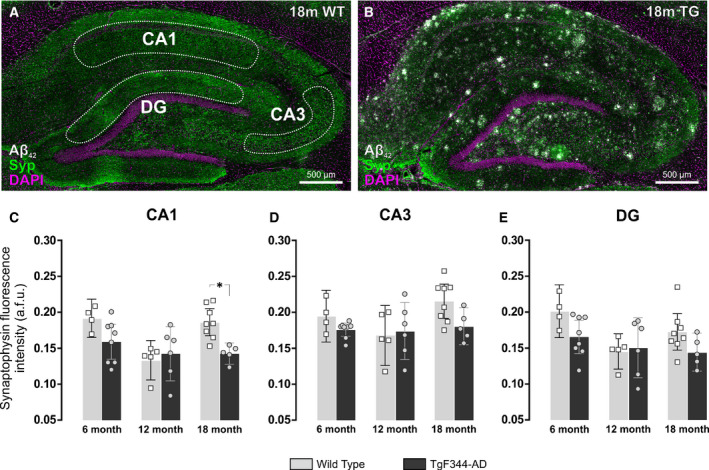
Synaptophysin expression decreases in the hippocampal CA1 region of 18‐month TgF344‐AD rats. (A) Sagittal section of the dorsal hippocampus of an 18‐month wild‐type (WT) rat and (B) 18‐month TgF344‐AD (TG) rat immunofluorescently labelled using the cell nucleus marker DAPI (magenta), the amyloid plaque marker Aβ_1‐42_ (white), and the presynaptic marker synaptophysin (Syp; green). Scale bar = 500 µm. (C) There were no significant differences in synaptophysin expression between WT and TG rats at 6 and 12 months of age. However, 18‐month TG rats presented with lower levels of synaptophysin staining at 18‐months compared to age‐matched WT controls. Conversely, there were no significant changes in synaptophysin expression with age or amyloid plaque accumulation in the (D) CA3 and (E) dentate gyrus (DG) regions. Data are presented as the mean ± 95% confidence intervals and analysed using a two‐way ANOVA with Holm‐Sidak post hoc test. * represents a *P* value < 0.05. The number (n) of rats per group was as follows: 6m WT (*n* = 4); 12m WT (*n* = 5); 18m WT (*n* = 8); 6m TG (*n* = 8); 12m TG (*n* = 6); 18m TG (*n* = 5).

We next investigated if the expression of synaptophysin, a marker of the presynaptic bouton, changes with age in the CA1, CA3 or dentate gyrus of wild‐type and TgF344‐AD rats (Fig. 5A, B). Ageing had a significant effect on the intensity of synaptophysin staining in the molecular layer of the dentate gyrus, which is largely comprised of the dendrites of dentate granule cells (2‐way ANOVA, *F*_2, 30_ = 4.37, *P* = 0.022). However, there was no effect of genotype on synaptophysin expression in the dentate gyrus at any of the ages examined (Fig. 5E). Neither ageing nor genotype had any effect on synaptophysin expression in the CA3 region (Fig. 5D). However, both age (2‐way ANOVA, *F*_2, 30_ = 6.26, *P* = 0.005) and genotype (2‐way ANOVA, *F*_1, 30_ = 6.55, *P* = 0.016) had a significant effect on synaptophysin levels in the stratum pyramidale and stratum radiatum of the CA1 region (Fig. 5C), which are composed of the cell bodies and axons of CA1 pyramidal neurons, respectively. Moreover, there was an interaction between age and genotype in the CA1 region, implying that the effects of age on synaptophysin levels depend on the genotype of the rat (2‐way ANOVA, *F*_2, 30_ = 3.37, *P* = 0.048). As such, a decrease in synaptophysin expression was measured in the CA1 region of 18‐month TgF344‐AD rats compared to 18‐month wild‐types (mean = 0.19, 95% CI = [0.17, 0.21] vs 0.14, 95% CI = [0.13, 0.16] a.f.u., *P* = 0.016). Therefore, CA1 pyramidal neurons of transgenic rats appeared vulnerable to synapse loss at 18‐months of age. However, there were no significant differences in synaptophysin expression between 18‐month TgF344‐AD rats compared to their 12‐month (*P* = 1.0) and 6‐month (*P* = 0.990) counterparts and, in all cases, transgenic rats displayed a lower mean synaptophysin intensity with respect to the 18‐month wild‐type rats (18m WT mean = 0.19, 95% CI = [0.17, 0.21]; 18m TG mean = 0.14, 95% CI = [0.13, 0.16]; 12m TG mean = 0.14, 95% CI = [0.10, 0.18]; 6m TG mean = 0.16, 95% CI = [0.13, 0.18]).

### Hippocampal axonal density decreases with age in wild‐type and TgF344‐AD rats

**Fig. 6:**
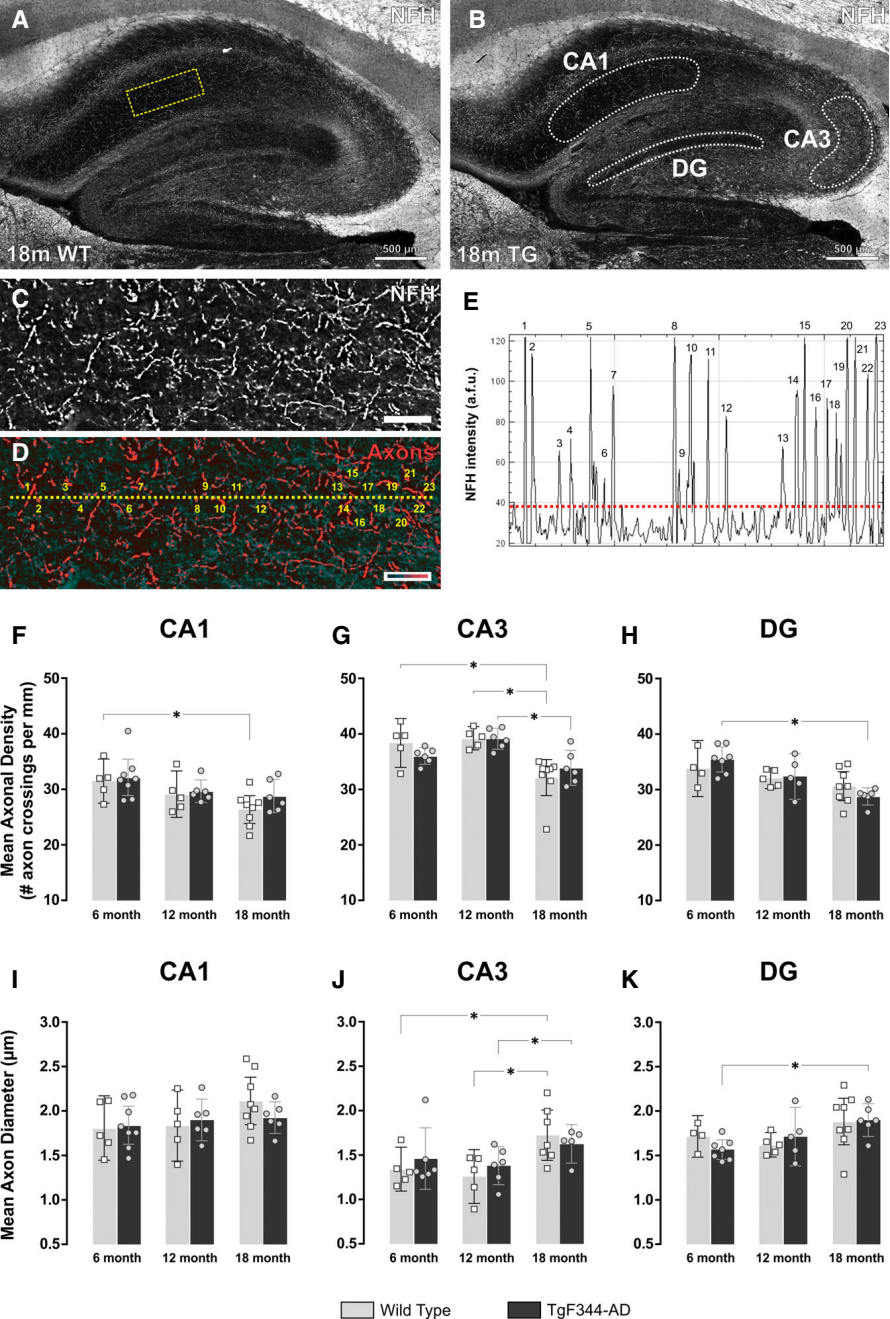
Ageing impacts neuronal density and axonal diameter in the hippocampus of wild‐type and TgF344‐AD rats. (A) Sagittal section of the dorsal hippocampus of an 18‐month wild‐type (WT) rat and (B) 18‐month TgF344‐AD (TG) rat immunofluorescently‐labelled using the axonal marker neurofilament‐H (NFH; white). Scale bar = 500 µm. (C) Cropped image showing the area delineated by the yellow box in (A). A pseudo‐colour palette in (D) highlights the neuronal axons (red) from background NFH staining (teal). Scale bar = 75 µm. The broken yellow line in (D) corresponds to the broken red line in (E). It transects 23 neuronal axons in the stratum radiatum of the CA1 region of the hippocampus. Axonal density was measured by calculating the mean number of axons per mm in the CA1, CA3 and dentate gyrus (DG). The mean axonal diameter was measured by calculating the width at half the height of the peak of NFH fluorescence intensity, as described in [27]. (F–H) There was a decrease in axonal density in the CA1 and CA3 regions from 6 to 18 months in WT rats. There were also decreases in axonal density in the CA3 region of TgF344‐AD rats from 12 to 18 months, and in the dentate gyrus from 6 to 18 months of age. (I–K) There were no changes in axonal diameter in the CA1 region of WT or TG rats. However, there were increases in axonal diameter in the CA3 region from 12 to 18 months of age in both WT and TG rats. Moreover, there was an increase in axonal diameter from 6 to 18 months in the dentate gyrus of TG rats. Data are presented as the mean ± 95% confidence intervals and analysed using a two‐way ANOVA with Holm‐Sidak post hoc test. *Represents a *P* value < 0.05. The number (*n*) of rats per group were as follows; 6m WT (*n* = 5); 12m WT (*n* = 5); 18m WT (*n* = 8); 6m TG (*n* = 8); 12m TG (*n* = 6); 18m TG (*n* = 6).

Since 18‐month TgF344‐AD rats displayed lower synapse density (measured by synaptophysin staining) in the CA1 region of the hippocampus, we next measured expression of the neuronal cytoskeletal intermediate filament protein, neurofilament heavy chain (NFH). When phosphorylated, NFH is involved in the maintenance of axonal calibre. Hippocampal axonal density was calculated by counting the number of axon crossings per unit length in the stratum radiatum of the CA1, the stratum pyramidale of the CA3 and the granule cell layer of the dentate gyrus (Fig. 6A, B). Axonal diameter was also calculated as described in [27]. Figure 6C–E illustrates a typical neuronal density trace from the CA1 region of the hippocampus. Interestingly, there were no differences in axonal density at 6, 12 or 18 months between wild‐type and TgF344‐AD rats (Fig. 6F–H). Similarly, there were no differences in axonal diameter between wild‐type and TgF344‐AD rats at 6, 12 or 18 months (Fig. 6I–K). However, ageing had the largest effect on axonal diameter and density. In the CA1 region, axonal density decreased from 6 to 18 months in wild‐types (mean = 31.47, 95% CI = [27.47, 35.46] vs 26.35, 95% CI = [23.85, 28.84], *P* = 0.016) but not transgenic rats (Fig. 6F). In the dentate gyrus, axonal density decreased from 6 to 18 months in transgenic rats (mean = 35.44, 95% CI = [33.17, 37.71] vs 28.75, 95% CI = [27.20, 30.30], *P* = 0.007), but not in wild‐types (Fig. 6H). This was accompanied by an age‐related increase in axonal diameter in the DG of TgF344‐AD rats (Fig. 6K). The CA3 region also demonstrated age‐associated decreases in axonal density (Fig. 6G) and increases in axonal diameter (Fig. 6J) in both wild‐type and TgF344‐AD rats.

### Ageing and amyloid burden increase reactive astrogliosis in the hippocampus of wild‐type and TgF344‐AD rats

**Fig. 7:**
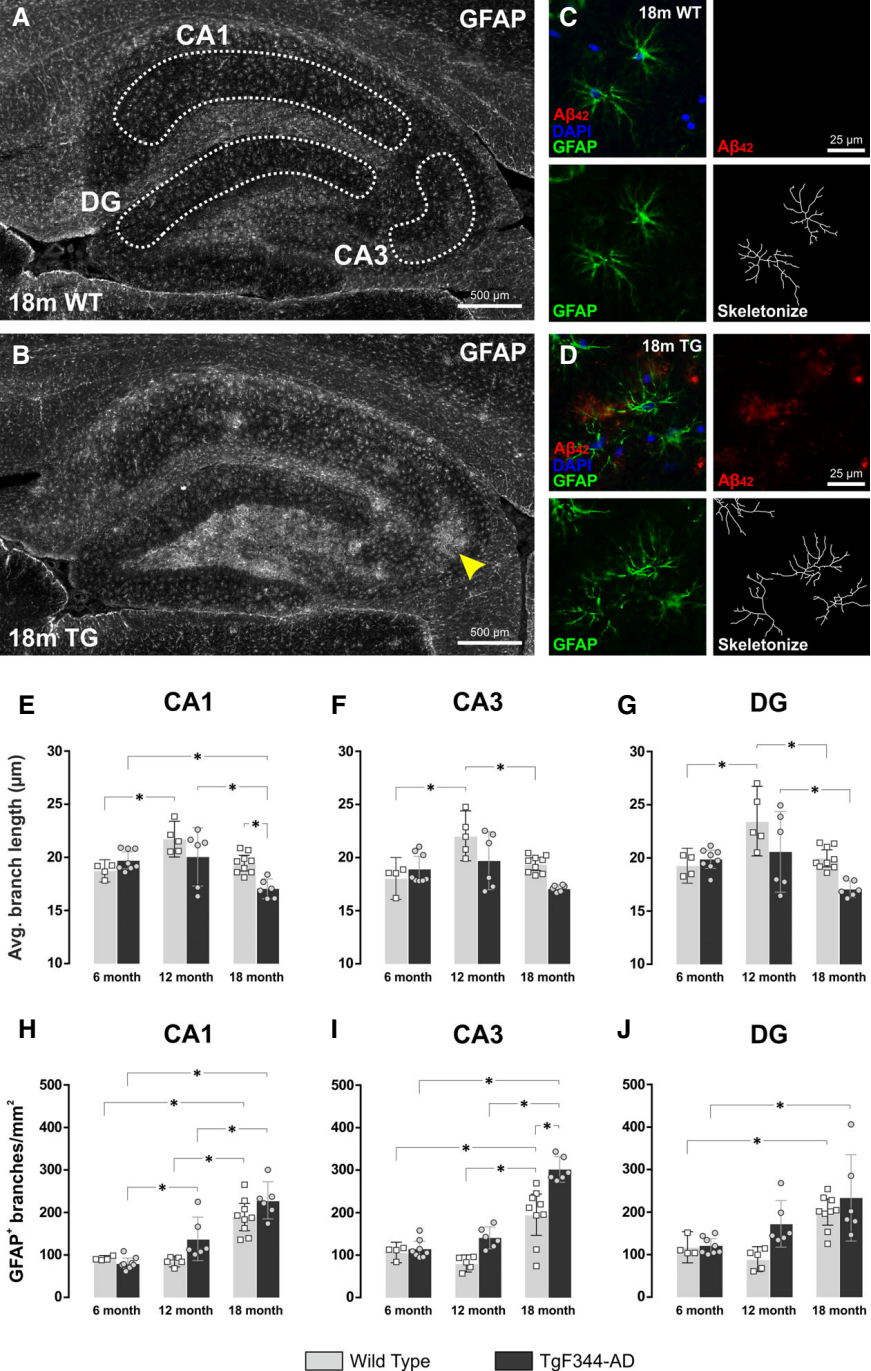
Hippocampal astrocyte morphology in wild‐type and TgF344‐AD rats. (A) Sagittal section of the dorsal hippocampus of an 18‐month wild‐type (WT) rat and (B) 18‐month TgF344‐AD (TG) rat immunofluorescently labelled using the astrocyte marker GFAP (white). Scale bar = 500 µm. (C–D) Cropped images of the CA1 region of (C) an 18m WT rat and (D) an 18m TG rat, immunofluorescently labelled with DAPI (blue), Aβ_1‐42_ (red) and GFAP (green). Scale bar = 25 µm. Astrocyte morphology was measured by converting the GFAP fluorescence to a binary image and using the *Skeletonize* tool in ImageJ software. The average branch length (in µm) and astrocyte density (measured as number of skeletons per unit area) was calculated in the hippocampal regions depicted in (A). (E–G) There were increases in astrocytic branch length in all hippocampal regions of WT rats from 6 to 12 months of age. However, astrocyte branch length decreased to 6‐month levels in aged (18‐month) WT rats. Astrocyte branch length decreased even further in 18‐month TG rats. (H–J) There was an increase in the mean number of astrocyte (GFAP‐positive) branches in both WT and TG rats from 6 to 18 months of age. This could represent either (or both) an increase in the number of branches per astrocyte or an increase in the total number of astrocytes per unit area. There was a particularly large increase in astrocyte branch number in the CA3 region of 18m TG rats (i.e., yellow arrow in B). Data are presented as the mean ± 95% confidence intervals and analysed using a 2‐way ANOVA with Holm‐Sidak post hoc test. *Represents a *P* value < 0.05. The number (n) of rats per group was as follows: 6m WT (*n* = 4); 12m WT (*n* = 5); 18m WT (*n* = 9); 6m TG (*n* = 8); 12m TG (*n* = 6); 18m TG (*n* = 6).

Finally, we measured changes in glial fibrillary acidic protein (GFAP) expression and astrocyte morphology in the hippocampus of ageing wild‐type and TgF344‐AD rats (Fig. 7A–D). The hippocampus was subdivided into the stratum radiatum of the CA1, the stratum lucidum and pyramidal layer of the CA3, and the molecular layer of the dentate gyrus. Interestingly, the average length of astrocyte processes increased from 6 to 12 months in wild‐type, but not TgF344‐AD rats (Fig. 7E–G). However, astrocyte branch length then decreased from 12 to 18 months in wild‐type rats (Fig. 7F, G). In the CA1 region, 18‐month TgF344‐AD rats had significantly shorter astrocytic processes to 18‐month wild‐types (mean = 17.03 µm, 95% CI = [16.10, 17.97] vs 19.45 µm, 95% CI = [18.71, 20.18], *P* = 0.026) (Fig. 7E). Ageing also impacted the number of astrocytic processes counted per unit area. Both wild‐type and TgF344‐AD rats displayed an increase in the number of astrocyte processes from 6 to 18 months in all hippocampal areas examined (Fig. 7H–J). This most likely reflects an increase in the complexity of individual astrocytes (i.e., a decrease in branch length and increase in branch number). However, it may also reflect a concomitant increase in astrocyte numbers (i.e., astrocyte density). There was a striking increase in GFAP expression in the hilus region of the dentate gyrus as well as around amyloid plaques (Fig. 7B, yellow arrow). As such, there was a significant increase in astrocyte branch number in the CA3 region of TgF344‐AD rats versus wild‐types (mean = 301.6, 95% CI = [271.7, 331.5] vs 195.1, 95% CI = [146.7, 243.5], p < 0.0001) (Fig. 7I).

## Discussion

The transcription factor, REST, was initially recognised for its important role in neurogenesis [32]. As a master regulator of neuronal gene expression, it also plays a key role in shaping the function and plasticity of mature neurons [33]. Given the wide variety of neuron‐specific REST gene targets [34], it has recently gained recognition as a modulator of neuronal homeostasis in both healthy brain ageing and neurodegenerative disease contexts [35, 36]. However, detailed mechanistic insight into how REST regulates target gene expression is still lacking, mainly because of the large number of candidate genes and the complexity in deciphering REST’s functional role in their transcription or repression [17, 37, 38]. For example, REST is known to repress glucocorticoid‐mediated induction of glutamine synthetase (GS) expression in non‐neuronal cells. Conversely, a splice variant of REST (i.e., REST4) may enhance GS expression in neurons [39]. Therefore, depending on the context and the gene target, REST can act as either a repressor or activator of gene transcription [40]. Moreover, it has been suggested that an increase in REST in cortical neurons may combat the potentially harmful effects of sustained enhancements in neuronal firing and elevated glutamatergic synaptic transmission [41, 42]. Indeed, an increase in neuronal activity, as measured by fMRI, has been observed in the prefrontal cortex of aged individuals [43]. However, other studies have shown that REST activity may be a biomarker of neuronal senescence [44, 45]. Therefore, REST activity may be enhanced in response to stressful cellular conditions, but once elevated, REST can exert either protective or damaging functions, depending on the context [17].

In this study, we show for the first time the spatiotemporal expression profile of nuclear REST throughout the healthy ageing brain and the brain of Alzheimer’s disease rats. Here, nuclear REST expression increases in the frontal cortex from 6 to 18 months, but not in more posterior regions of the neocortex. Interestingly, we found no significant differences in the levels of cortical REST between wild‐type and TgF344‐AD rats. This is in contrast to the study by Lu et al. [13] who demonstrated a loss of nuclear REST in postmortem prefrontal cortex tissue from human AD patients in comparison with healthy age‐matched controls. However, even though the expected downregulation of nuclear REST expression did not occur in the neocortex of TgF344‐AD rats, there were also no significant increases in REST from 6 to 18 months of age. Therefore, if nuclear translocation of REST is indeed protective in healthy ageing cortical neurons [36], then failure of neurons to upregulate nuclear REST in TgF344‐AD rats could be equally detrimental in the context of neurodegenerative disease. In Alzheimer’s disease, oxidative stress activates neuronal autophagosomes which can engulf REST together with other misfolded proteins, such as Aβ and tau [13]. As a result, the translocation of REST to the nucleus may be reduced. In human Parkinson’s disease brains, REST protein has been found within cytoplasmic protein aggregates suggesting that protein inclusions, such as Lewy bodies, can sequester the protein and prevent nuclear translocation [16]. However, we did not examine the potential sequestration of REST protein within the amyloid plaques found in the TgF344‐AD rats.

The hippocampus is particularly vulnerable to damage caused by ischaemic insults, chronic stress, and neurodegenerative diseases [46, 47]. In wild‐type animals, we found that ageing increased nuclear REST expression in the hippocampus and subiculum, which is the main output structure of the hippocampus and receives input from CA1 pyramidal neurons [48]. Interestingly, the subiculum relays inhibitory signals from the hippocampus to the hypothalamic‐pituitary axis (HPA) to dampen stress responses [49]. Therefore, age‐related nuclear translocation of REST may be more common in brain regions actively involved in regulating the response to stress. Again, in our study, REST nuclear translocation did not occur in the subiculum or hippocampus (CA1, CA3 and DG) of TgF344‐AD rats with advancing age. In contrast, Lu et al. [13] reported reduced levels of nuclear REST exclusively in neurons of the prefrontal cortex and hippocampal neurons (CA1, CA3 and CA4, but not DG) in the human AD brain. The levels of nuclear REST were not altered in neurons of the cerebellum, which are less affected by the classical neuropathological hallmarks of AD [13, 50]. Therefore, the brain regions that displayed the most significant increases in nuclear REST in ageing wild‐type rats in the present study, correspond to structures that are strongly impacted by amyloid plaque pathology [13]. Thus, if elevations in nuclear REST are a protective mechanism to defend neurons against Aβ_1–42_ mediated toxicity, then the inability of cortical and hippocampal neurons to increase nuclear REST in the TgF344‐AD rat model likely represents a blunted response to pathological challenges and could be equally detrimental as a decrease in REST expression.

Ageing neurons possess larger mitochondria and undergo structural alterations, including an increase in axonal diameter [51]. These signs of ageing are often correlated with increased generation of reactive oxygen species (ROS) and a disruption to cellular Ca^2+^ homeostasis, which may induce increases in nuclear REST [51, 52]. Here, increases in hippocampal REST expression in wild‐type rats from 6 to 18 months coincided with a decrease in axonal density in both the CA1 and CA3 regions, and an increase in axonal diameter, specifically in the CA3 region. Several studies have shown that ageing coincides with a decrease in axonal density in the hippocampus [51, 53]. However, the elevated nuclear REST we observed in the CA1 pyramidal cell layer did not protect against axonal loss in the CA1 stratum radiatum. Nevertheless, augmented levels of nuclear REST may have helped to protect 18‐month wild‐type CA1 neurons from a loss of synapses.

We show here that TgF344‐AD rats display a significant decrease in the presynaptic marker, synaptophysin (Syp), in CA1 neurons at 18‐months of age. At first, this result appears counterintuitive because the Syp gene is a target of REST‐mediated transcriptional repression in non‐neuronal cells and high levels of REST activity usually repress the expression of classic neuronal markers, such as the synaptophysin protein [54]. However, elevated REST expression in wild‐type CA1 neurons may instead function to silence stress‐ and cell death‐associated genes, for example members of the Notch signalling pathway [55, 56], and thus could help to protect wild‐type neurons from Aβ_1–42_ mediated loss of synapses indirectly [35]. Indeed, dynamic and selective regulation of REST target genes plays an important role in modulating neuronal properties in both physiological and pathological contexts [14]. In humans with AD, increased immunoreactivity for the apoptosis marker FADD has been shown in cholinergic neurons of the nucleus basalis of Meynert [57]. Moreover, neuronal progenitor cells derived from sporadic AD induced pluripotent stem cells (iPSCs) were shown to have reduced levels of nuclear REST in comparison to iPSCs derived from normal controls [58]. This lower expression level of nuclear REST in sporadic AD iPSCs was linked to decreased binding of REST to the RE1 motif of target genes, such as ASCL1, CALB1, DCX, STMN2 and SNAP25, which led to premature neuronal differentiation, accelerated synapse formation and increased excitability [58]. Thus, failure to upregulate REST in the TgF344‐AD rat brain may constitute signs of blunted neuroprotective homeostatic mechanisms and increased vulnerability to molecular stressors [13, 15, 16]. Of note, Meyer et al. [58] suggested that the magnitude of the reduction in REST docking to the RE1 binding site within its target gene chromatin is just as functionally significant as the overall reduction in nuclear REST levels.

The increase in hippocampal REST expression in wild‐type rats from 6 to 18 months of age also coincided with an increase in astrogliosis. However, quantification of REST expression was performed in the pyramidal and granule neuronal cell layers and therefore, an increase of nuclear REST predominantly within neurons would not necessarily prevent glial reactivity in old age. It has been shown that primary cultured astrocytes from astrocyte‐specific REST conditional knockout mice display an enhanced inflammatory response to MPP+ (1‐methyl‐4‐phenylpyridinium) and LPS (lipopolysaccharide) [59]. Moreover, the inability to recruit REST to the nucleus in response to the neurotoxin MPTP (1‐methyl‐4‐phenyl‐1,2,3,6‐tetrahydropyridine) is also detrimental in neuron‐specific REST conditional knockout mice and results in elevated levels of the pro‐inflammatory cytokine, IL‐1β, and increases in GFAP expression in astrocytes [60]. This is interesting because TgF344‐AD rats did not show elevated nuclear REST in response to ageing, and yet transgenic animals displayed higher levels of astroglial reactivity and hypertrophy in the hippocampus, particularly in the CA3 and hilar (CA4) regions. This suggests an interplay between neuron‐ and glial‐specific REST regulation.

## Conclusions

We have shown that nuclear REST increases with healthy ageing in the frontal cortex, subiculum and hippocampus of wild‐type rats. These brain areas are important for learning, memory formation, language and higher cognitive functions and are those most affected by age‐related brain atrophy and neurodegeneration [61, 62]. REST expression, however, does not change with age in the TgF344‐AD rat model of Alzheimer’s disease. There is evidence to suggest that neuroinflammation and astrogliosis may be more pronounced in TgF344‐AD rats compared to wild‐type Fischer‐344 rats. This dampened REST response in the TgF344‐AD rat brain may be detrimental. Moreover, based on what is currently known about the functions of REST in various disease contexts, failure to upregulate nuclear REST activity could prevent key cellular stress responses from being activated as Aβ_1–42_ plaques gradually accumulate and neurodegenerative signals increase in the Alzheimer’s disease brain. In conclusion, our results raise several questions that warrant further investigation: (1) Is REST binding affinity to RE1 binding sites within target gene chromatin dependent on the brain region analysed or is it possibly age‐ and/or disease‐specific? (2) How are neighbouring glial cells affected by the failure of neurons to upregulate REST in the context of Alzheimer’s disease pathogenesis?

## Conflict of interest

The authors declare no conflict of interest.

## Author contributions

GKS and HB conceived the project and designed the research. MVE, AMC, HB and GKS performed the experiments. MVE, MM, SOR and GKS analysed the data. GKS prepared the figures. All authors discussed the data and interpreted the results. GKS, MM and MVE wrote the manuscript with intellectual input and critical revisions from all authors. All authors reviewed and approved the final draft of the manuscript.

## Supporting information


**Fig S1.** Overview of the image analysis technique used to measure changes in nuclear REST expression in the rat brain.

## Data Availability

The data that accompany this manuscript are available from the corresponding author upon reasonable request.

## References

[1] Lansdall CJ , Coyle‐Gilchrist ITS , Jones PS , Vázquez Rodríguez P , Wilcox A , Wehmann E , Dick KM , Robbins TW and Rowe JB (2017) Apathy and impulsivity in frontotemporal lobar degeneration syndromes. Brain 140, 1792–1807. 28486594 10.1093/brain/awx101 PMC5868210

[2] Lane‐Donovan C and Herz J (2017) ApoE, ApoE receptors, and the synapse in Alzheimer’s disease. Trends Endocrinol Metab 28, 273–284. 28057414 10.1016/j.tem.2016.12.001 PMC5366078

[3] Xia X , Jiang Q , McDermott J and Han J‐DJ (2018) Aging and Alzheimer’s disease: comparison and associations from molecular to system level. Aging Cell 17, e12802. 29963744 10.1111/acel.12802 PMC6156542

[4] Querfurth HW and LaFerla FM (2010) Alzheimer’s Disease. N Engl J Med 362, 329–344. 20107219 10.1056/NEJMra0909142

[5] Bertoni‐Freddari C , Fattoretti P , Casoli T , Caselli U and Meier‐Ruge W (1996) Deterioration threshold of synaptic morphology in aging and senile dementia of Alzheimer’s type. Anal Quant Cytol Histol 18, 209–213. 8790834

[6] DeKosky ST and Scheff SW (1990) Synapse loss in frontal cortex biopsies in Alzheimer’s disease: correlation with cognitive severity. Ann Neurol 27, 457–464. 2360787 10.1002/ana.410270502

[7] Selkoe DJ (2002) Alzheimer’s disease is a synaptic failure. Science 298, 789–791. 12399581 10.1126/science.1074069

[8] Jackson J , Jambrina E , Li J , Marston H , Menzies F , Phillips K and Gilmour G (2019) Targeting the synapse in Alzheimer’s disease. Front Neurosci 13, 735. 31396031 10.3389/fnins.2019.00735 PMC6664030

[9] Scheff SW , Price DA , Schmitt FA and Mufson EJ (2006) Hippocampal synaptic loss in early Alzheimer’s disease and mild cognitive impairment. Neurobiol Aging 27, 1372–1384. 16289476 10.1016/j.neurobiolaging.2005.09.012

[10] Yankner BA , Lu T and Loerch P (2008) The aging brain. Annu Rev Pathol Mech Dis 3, 41–66. 10.1146/annurev.pathmechdis.2.010506.092044 18039130

[11] Akiyama H (2000) Inflammation and Alzheimer’s disease. Neurobiol Aging 21, 383–421. 10858586 10.1016/s0197-4580(00)00124-x PMC3887148

[12] Heneka MT , Carson MJ , Khoury JE , Landreth GE , Brosseron F , Feinstein DL , Jacobs AH , Wyss‐Coray T , Vitorica J , Ransohoff RM *et al* (2015) Neuroinflammation in Alzheimer’s disease. Lancet Neurol 14, 388–405. 25792098 10.1016/S1474-4422(15)70016-5 PMC5909703

[13] Lu T , Aron L , Zullo J , Pan Y , Kim H , Chen Y , Yang T‐H , Kim H‐M , Drake D , Liu XS *et al* (2014) REST and stress resistance in ageing and Alzheimer’s disease. Nature 507, 448–454. 24670762 10.1038/nature13163 PMC4110979

[14] McClelland S , Brennan GP , Dubé C , Rajpara S , Iyer S , Richichi C , Bernard C and Baram TZ (2014) The transcription factor NRSF contributes to epileptogenesis by selective repression of a subset of target genes. eLife 3, e01267. 25117540 10.7554/eLife.01267 PMC4129437

[15] Dallagnol KMC , Remor AP , da Silva RA , Prediger RD , Latini A and Aguiar AS (2017) Running for REST: Physical activity attenuates neuroinflammation in the hippocampus of aged mice. Brain Behav Immun 61, 31–35. 27477921 10.1016/j.bbi.2016.07.159

[16] Kawamura M , Sato S , Matsumoto G , Fukuda T , Shiba‐Fukushima K , Noda S , Takanashi M , Mori N and Hattori N (2019) Loss of nuclear REST/NRSF in aged‐dopaminergic neurons in Parkinson’s disease patients. Neurosci Lett 699, 59–63. 30684677 10.1016/j.neulet.2019.01.042

[17] Mampay M and Sheridan GK (2019) REST: An epigenetic regulator of neuronal stress responses in the young and ageing brain. Front Neuroendocrinol 53, 100744. 31004616 10.1016/j.yfrne.2019.04.001

[18] Zuccato C , Tartari M , Crotti A , Goffredo D , Valenza M , Conti L , Cataudella T , Leavitt BR , Hayden MR , Timmusk T *et al* (2003) Huntingtin interacts with REST/NRSF to modulate the transcription of NRSE‐controlled neuronal genes. Nat Genet 35, 76–83. 12881722 10.1038/ng1219

[19] DiSabato DJ , Quan N and Godbout JP (2016) Neuroinflammation: the devil is in the details. J Neurochem 139, 136–153. 26990767 10.1111/jnc.13607 PMC5025335

[20] Cohen RM , Rezai‐Zadeh K , Weitz TM , Rentsendorj A , Gate D , Spivak I , Bholat Y , Vasilevko V , Glabe CG , Breunig JJ *et al* (2013) A transgenic Alzheimer rat with plaques, Tau pathology, behavioral impairment, oligomeric A, and frank neuronal loss. J Neurosci 33, 6245–6256. 23575824 10.1523/JNEUROSCI.3672-12.2013 PMC3720142

[21] Schindelin J , Arganda‐Carreras I , Frise E , Kaynig V , Longair M , Pietzsch T , Preibisch S , Rueden C , Saalfeld S , Schmid B *et al* (2012) Fiji: an open‐source platform for biological‐image analysis. Nat Methods 9, 676–682. 22743772 10.1038/nmeth.2019 PMC3855844

[22] Sbalzarini IF and Koumoutsakos P (2005) Feature point tracking and trajectory analysis for video imaging in cell biology. J Struct Biol 151, 182–195. 16043363 10.1016/j.jsb.2005.06.002

[23] Velasco‐Estevez M , Mampay M , Boutin H , Chaney A , Warn P , Sharp A , Burgess E , Moeendarbary E , Dev KK and Sheridan GK (2018) Infection augments expression of mechanosensing piezo1 channels in amyloid plaque‐reactive astrocytes. Front Aging Neurosci 10, 332. 30405400 10.3389/fnagi.2018.00332 PMC6204357

[24] McQuin C , Goodman A , Chernyshev V , Kamentsky L , Cimini BA , Karhohs KW , Doan M , Ding L , Rafelski SM , Thirstrup D *et al* (2018) Cell Profiler 3.0: Next‐generation image processing for biology. PLoS Biol 16, e2005970. 29969450 10.1371/journal.pbio.2005970 PMC6029841

[25] Schneider CA , Rasband WS and Eliceiri KW (2012) NIH Image to ImageJ: 25 years of image analysis. Nat Methods 9, 671–675. 22930834 10.1038/nmeth.2089 PMC5554542

[26] Meijering E (2003) FeatureJ: A Java Package for Image Feature Extraction. https://imagescience.org/meijering/software/featurej/

[27] Sathyanesan A , Ogura T and Lin W (2012) Automated measurement of nerve fiber density using line intensity scan analysis. J Neurosci Methods 206, 165–175. 22613744 10.1016/j.jneumeth.2012.02.019 PMC3358701

[28] Doube M , Kłosowski MM , Arganda‐Carreras I , Cordelières FP , Dougherty RP , Jackson JS , Schmid B , Hutchinson JR and Shefelbine SJ (2010) BoneJ: Free and extensible bone image analysis in ImageJ. Bone 47, 1076–1079. 20817052 10.1016/j.bone.2010.08.023 PMC3193171

[29] Gomez‐Arboledas A , Davila JC , Sanchez‐Mejias E , Navarro V , Nuñez‐Diaz C , Sanchez‐Varo R , Sanchez‐Mico MV , Trujillo‐Estrada L , Fernandez‐Valenzuela JJ , Vizuete M *et al* (2018) Phagocytic clearance of presynaptic dystrophies by reactive astrocytes in Alzheimer’s disease. Glia 66, 637–653. 29178139 10.1002/glia.23270 PMC5814816

[30] Pomilio C , Pavia P , Gorojod RM , Vinuesa A , Alaimo A , Galvan V , Kotler ML , Beauquis J and Saravia F (2016) Glial alterations from early to late stages in a model of Alzheimer’s disease: evidence of autophagy involvement in Aβ internalization: neuroinflammation progress in a model of AD. Hippocampus 26, 194–210. 26235241 10.1002/hipo.22503 PMC5467976

[31] Osborn LM , Kamphuis W , Wadman WJ and Hol EM (2016) Astrogliosis: An integral player in the pathogenesis of Alzheimer’s disease. Prog Neurogibol 144, 121–141. 10.1016/j.pneurobio.2016.01.001 26797041

[32] Schoenherr C and Anderson D (1995) The neuron‐restrictive silencer factor (NRSF): a coordinate repressor of multiple neuron‐specific genes. Science 267, 1360–1363. 7871435 10.1126/science.7871435

[33] Rodenas‐Ruano A , Chávez AE , Cossio MJ , Castillo PE and Zukin RS (2012) REST‐dependent epigenetic remodeling promotes the developmental switch in synaptic NMDA receptors. Nat Neurosci 15, 1382–1390. 22960932 10.1038/nn.3214 PMC3501125

[34] Bruce AW , Donaldson IJ , Wood IC , Yerbury SA , Sadowski MI , Chapman M , Gottgens B and Buckley NJ (2004) Genome‐wide analysis of repressor element 1 silencing transcription factor/neuron‐restrictive silencing factor (REST/NRSF) target genes. Proc Natl Acad Sci USA 101, 10458–10463. 15240883 10.1073/pnas.0401827101 PMC478591

[35] Hwang J‐Y and Zukin RS (2018) REST, a master transcriptional regulator in neurodegenerative disease. Curr Opin Neurobiol 48, 193–200. 29351877 10.1016/j.conb.2017.12.008 PMC5892838

[36] Baldelli P and Meldolesi J (2015) The transcription repressor REST in adult neurons: physiology, pathology, and diseases. eneuro 2, ENEURO.0010‐15.2015. 10.1523/ENEURO.0010-15.2015 PMC4596026 26465007

[37] Song Z , Zhao D , Zhao H and Yang L (2015) NRSF: an angel or a devil in neurogenesis and neurological diseases. J Mol Neurosci 56, 131–144. 25479824 10.1007/s12031-014-0474-5

[38] Zhao Y , Zhu M , Yu Y , Qiu L , Zhang Y , He L and Zhang J (2017) Brain REST/NRSF is not only a silent repressor but also an active protector. Mol Neurobiol 54, 541–550. 26742529 10.1007/s12035-015-9658-4

[39] Abramovitz L , Shapira T , Ben‐Dror I , Dror V , Granot L , Rousso T , Landoy E , Blau L , Thiel G and Vardimon L (2008) Dual role of NRSF/REST in activation and repression of the glucocorticoid response. J Biol Chem 283, 110–119. 17984088 10.1074/jbc.M707366200

[40] Roopra A , Huang Y and Dingledine R (2001) Neurological disease: listening to gene silencers. Mol Interv 1, 219–228. 14993344

[41] Pozzi D , Lignani G , Ferrea E , Contestabile A , Paonessa F , D’Alessandro R , Lippiello P , Boido D , Fassio A , Meldolesi J *et al* (2013) REST/NRSF‐mediated intrinsic homeostasis protects neuronal networks from hyperexcitability: hyperexcitability induces REST‐mediated intrinsic homeostasis. EMBO J 32, 2994–3007. 24149584 10.1038/emboj.2013.231 PMC3831314

[42] Hu X‐L , Cheng X , Cai L , Tan G‐H , Xu L , Feng X‐Y , Lu T‐J , Xiong H , Fei J and Xiong Z‐Q (2011) Conditional deletion of NRSF in forebrain neurons accelerates epileptogenesis in the kindling model. Cereb Cortex 21, 2158–2165. 21339379 10.1093/cercor/bhq284

[43] Morcom AM and Henson RNA (2018) Increased prefrontal activity with aging reflects nonspecific neural responses rather than compensation. J Neurosci Off J Soc Neurosci 38, 7303–7313. 10.1523/JNEUROSCI.1701-17.2018 PMC6096047 30037829

[44] Ishikawa S and Ishikawa F . (2020) Proteostasis failure and cellular senescence in long‐term cultured postmitotic rat neurons. Aging Cell 19, e13071. 31762159 10.1111/acel.13071 PMC6974705

[45] Piechota M , Sunderland P , Wysocka A , Nalberczak M , Sliwinska MA , Radwanska K and Sikora E (2016) Is senescence‐associated β‐galactosidase a marker of neuronal senescence? Oncotarget 7, 81099–81109. 27768595 10.18632/oncotarget.12752 PMC5348379

[46] McEwen BS (1994) The plasticity of the hippocampus is the reason for its vulnerability. Semin Neurosci 6, 239–246.

[47] Michaelis EK (2012) Selective neuronal vulnerability in the hippocampus: relationship to neurological diseases and mechanisms for differential sensitivity of neurons to stress In The Clinical Neurobiology of the Hippocampus (Bartsch T, ed), pp. 54–76. Oxford University Press, Oxford, UK.

[48] Xu X , Sun Y , Holmes TC and López AJ (2016) Noncanonical connections between the subiculum and hippocampal CA1: noncanonical subicular connections. J Comp Neurol 524, 3666–3673. 27150503 10.1002/cne.24024 PMC5050062

[49] Mueller NK , Dolgas CM and Herman JP (2004) Stressor‐selective role of the ventral subiculum in regulation of neuroendocrine stress responses. Endocrinology 145, 3763–3768. 15142982 10.1210/en.2004-0097

[50] Jacobs HIL , Hopkins DA , Mayrhofer HC , Bruner E , van Leeuwen FW , Raaijmakers W and Schmahmann JD (2018) The cerebellum in Alzheimer’s disease: evaluating its role in cognitive decline. Brain 141, 37–47. 29053771 10.1093/brain/awx194

[51] Stahon KE , Bastian C , Griffith S , Kidd GJ , Brunet S and Baltan S (2016) Age‐related changes in axonal and mitochondrial ultrastructure and function in white matter. J Neurosci Off J Soc Neurosci 36, 9990–10001. 10.1523/JNEUROSCI.1316-16.2016 PMC5039264 27683897

[52] Ariano P , Zamburlin P , D’Alessandro R , Meldolesi J and Lovisolo D (2010) Differential repression by the transcription factor REST/NRSF of the various Ca2+ signalling mechanisms in pheochromocytoma PC12 cells. Cell Calcium 47, 360–368. 20171735 10.1016/j.ceca.2010.01.007

[53] Radhakrishnan H , Stark SM and Stark CEL (2020) Microstructural alterations in hippocampal subfields mediate age‐related memory decline in humans. Front Aging Neurosci 12, 94. 32327992 10.3389/fnagi.2020.00094 PMC7161377

[54] Lietz M , Hohl M and Thiel G (2002) RE‐1 silencing transcription factor (REST) regulates human synaptophysin gene transcription through an intronic sequence‐specific DNA‐binding site: regulation of synaptophysin gene transcription. Eur J Biochem 270, 2–9. 10.1046/j.1432-1033.2003.03360.x 12492469

[55] Aoki H , Ogino H , Tomita H , Hara A and Kunisada T (2016) Disruption of rest leads to the early onset of cataracts with the aberrant terminal differentiation of lens fiber cells. PLoS One 11, e0163042. 27631609 10.1371/journal.pone.0163042 PMC5025245

[56] Fischer DF (2005) Activation of the Notch pathway in Down syndrome: cross‐talk of Notch and APP. FASEB J 19, 1451–1458. 16126912 10.1096/fj.04-3395.com

[57] Wu C‐K , Thal L , Pizzo D , Hansen L , Masliah E and Geula C (2005) Apoptotic signals within the basal forebrain cholinergic neurons in Alzheimer’s disease. Exp Neurol 195, 484–496. 16085017 10.1016/j.expneurol.2005.06.020

[58] Meyer K , Feldman HM , Lu T , Drake D , Lim ET , Ling K‐H , Bishop NA , Pan Y , Seo J , Lin Y‐T *et al* (2019) REST and neural gene network dysregulation in iPSC models of Alzheimer’s disease. Cell Rep 26, 1112–1127.e9. 30699343 10.1016/j.celrep.2019.01.023 PMC6386196

[59] Li H , Liu Z , Wu Y , Chen Y , Wang J , Wang Z , Huang D , Wang M , Yu M , Fei J and Huang F . (2020) The deficiency of NRSF/REST enhances the pro‐inflammatory function of astrocytes in a model of Parkinson’s disease. Biochim Biophys Acta BBA – Mol Basis Dis 1866, 165590. 10.1016/j.bbadis.2019.165590 31706914

[60] Yu M , Suo H , Liu M , Cai L , Liu J , Huang Y , Xu J , Wang Y , Zhu C , Fei J *et al* (2013) NRSF/REST neuronal deficient mice are more vulnerable to the neurotoxin MPTP. Neurobiol Aging 34, 916–927. 22766071 10.1016/j.neurobiolaging.2012.06.002

[61] Hof PR , Glannakopoulos P and Bouras C (1996) The neuropathological changes associated with normal brain aging. Histol Histopathol 11, 1075–1088. 8930649

[62] Wegiel J , Flory M , Kuchna I , Nowicki K , Yong Ma S , Wegiel J , Badmaev E , Silverman WP , de Leon M , Reisberg B *et al* (2017) Multiregional age‐associated reduction of brain neuronal reserve without association with neurofibrillary degeneration or β‐amyloidosis. J Neuropathol Exp Neurol 76, 439–457. 28505333 10.1093/jnen/nlx027 PMC5901097

[63] Yu P , McKinney EC , Kandasamy MM , Albert AL and Meagher RB (2015) Characterization of brain cell nuclei with decondensed chromatin: decondensed brain cell nuclei. Dev Neurobiol 75, 738–756. 25369517 10.1002/dneu.22245

