# Supplementary material for: Spatiotemporal immunolocalisation of REST in the brain of healthy ageing and Alzheimer’s disease rats
Source: FEBS Open Bio. 2020 Dec 1;11(1):146–63. doi: 10.1002/2211-5463.13036 (PMC7780110; doi:10.1002/2211-5463.13036)
Supplement: Supplementary file 1 — Fig S1. Overview of the image analysis technique used to measure changes in nuclear REST expression in the rat brain. [file FEB4-11-146-s001.docx]

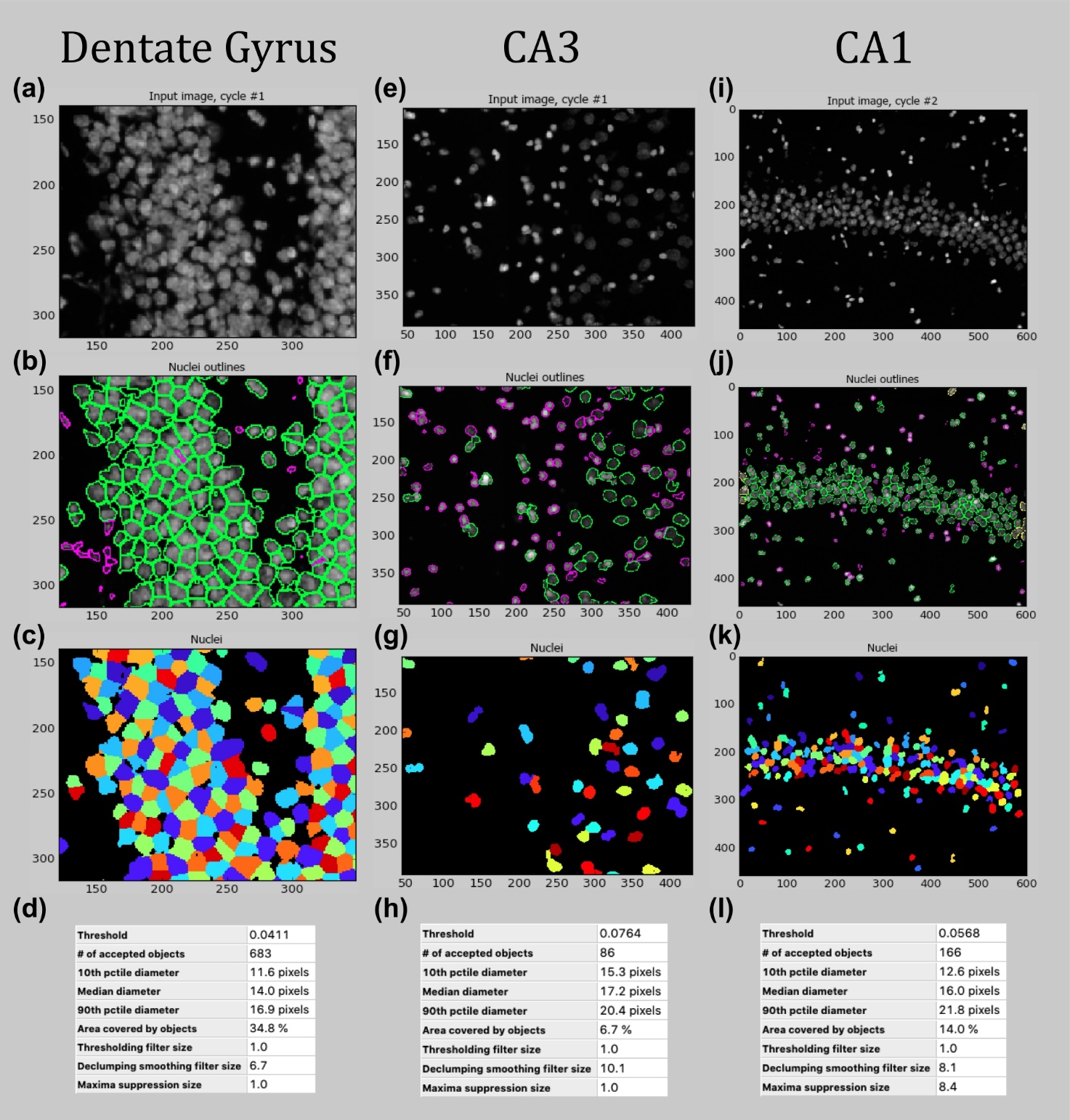


**Supplemental Figure 1:** **Overview of the image analysis technique used to measure changes in nuclear REST expression in the rat brain.** Cell Profiler™ was used to threshold DAPI-labelled nuclei in the brain and masks were generated for the automated analysis of REST fluorescence intensity within individual cell nuclei. Shown are example images of the **(a – d)** dentate gyrus, **(e – h)** CA3, and **(i – l)** CA1 regions of the hippocampus. Note, nuclei were chosen based on their size and diameter (no. of pixels) with larger green nuclei being chosen for REST quantification and smaller pink nuclei being excluded from the analysis. **(b, f, j)** All cell nuclei detected by the software and classified based on size. Note, the larger ‘green’ nuclei displayed faint DAPI staining indicating decondensed chromatin. This is particularly evident in images **(e)** and **(f)** of the CA3 region. These large DAPI-faint nuclei are usually NeuN-positive pyramidal cells whereas the small DAPI-intense nuclei are usually NeuN-negative (see Fig.1 in Sheridan et al., 2014; doi:10.3389/fncel.2014.00233). **(c, g, k)** The watershed cell nuclei selected for analysis.
